# What the Papers Say

**DOI:** 10.1093/jhps/hnv039

**Published:** 2015-06-17

The *Journal of Hip Preservation Surgery (JHPS)* is not the only place where work in the field of hip preservation may be published. Although our aim is to offer the best of the best, we continue to be fascinated by work that finds it way into journals other than our own. There is much to learn from it so *JHPS* has selected six recent and topical articles for those who seek a brief summary of what is taking place in our ever-fascinating world of hip preservation. What you see here are the mildly edited abstracts of the original articles, to give them what *JHPS* hopes is a more readable feel. Thanks to Ajay Malviya (UK), JHPS Editorial Correspondent, for his hard work in bringing this section together. If you are pushed for time, what follows should take you no more than 10 min to read. So here goes …

## Diagnostic performance of direct traction MR arthrography of the hip: detection of chondral and labral lesions with arthroscopic comparison[1]

Austrian researchers have assessed the diagnostic performance of traction MR arthrography of the hip in detection and grading of chondral and labral lesions with arthroscopic comparison.

Seventy-five MR arthrograms with or without traction of 73 consecutive patients who underwent hip arthroscopy were included. Traction technique included weight-adapted traction (15-23 kg), a supporting plate for the contralateral leg, and intra-articular injection of 18-27 ml (local anaesthetic and contrast agent). Two blinded readers independently assessed femoroacetabular cartilage and labrum lesions, which were correlated with arthroscopy. Interobserver agreement was calculated using Kappa (κ) values. Joint distraction with traction was evaluated in consensus.

Accuracy for detection was 92/93 % for labral lesions, 91/83 % for acetabular lesions, and 92/88 % for femoral cartilage lesions for reader 1/reader 2, respectively. Interobserver agreement was moderate (κ = 0.58) for grading of labrum lesions and substantial (κ = 0.7, κ = 0.68) for grading of acetabular and femoral cartilage lesions. Joint distraction was achieved in 72/75 and 14/75 hips with or without traction, respectively.

The authors concluded that traction MR arthrography safely enabled accurate detection and grading of labral and chondral lesions. The technique was well tolerated by most patients and consistently achieved separation of cartilage layers.

## Does Previous Pelvic Osteotomy Compromise the Results of Periacetabular Osteotomy Surgery?[2]

In a multi-centred North American study the authors assessed the outcome of Bernese periacetabular osteotomy (PAO) in patients after previous pelvic osteotomy. The purpose of this study was to compare the early pain, function, activity, and quality of life outcomes; radiographic correction; and major complications and failures between patients who underwent PAO after prior pelvic reconstruction versus those who had a PAO without prior surgery.

The study includes 39 patients who underwent PAO after prior pelvic osteotomy compared with a matched group of 78 patients without previous osteotomy with similar followup. Although both groups reached clinical improvement in all categorical measures, the revision PAO group demonstrated greater pain (HOOS pain, study 74 versus 85 control; p = 0.03) and less function (HOOS activities of daily living, study 80 versus 92 control; p = 0.002) than the primary cohort. The revision cohort achieved a smaller average radiographic correction than in patients undergoing PAO without prior pelvic surgery. The mean correction in acetabular inclination was less dramatic when directly comparing the revision and comparison groups (-12° to -17°; p < 0.001). Although there was no difference in severe complications requiring further surgery, there were two conversions to hip replacement (p = 0.109) in the study group.

The study concluded that although PAO performed after prior pelvic surgery is associated with improvements in pain, function, radiographic correction, and early complication rates, the improvements observed at short-term followup were smaller and more variable than those seen in patients who had not undergone prior pelvic surgery. The authors recommended that patients should be warned of potential ceiling effects with a second periacetabular surgery.

## Does Surgical Hip Dislocation and Periacetabular Osteotomy Improve Pain in Patients With Perthes-like Deformities and Acetabular Dysplasia?[3]

Research done in Washington has looked at hip preservation surgery for patients with symptomatic residual Perthes-like deformities. These patients present with a complex combination of structural abnormalities caused by concurrent symptomatic femoroacetabular impingement (proximal femoral deformities) and structural instability (acetabular dysplasia).

This study includes 16 patients with residual Perthes-like hip deformities and associated acetabular dysplasia treated with a combined surgical hip dislocation to comprehensively address intraarticular and extraarticular sources of FAI and PAO to address structural instability and were analysed at a minimum 24-month followup (range, 24-78 months).

Radiographic analysis demonstrated consistent radiographic correction. The median preoperative mHHS improved from 64 to 92 at a median followup of 40 months (p < 0.001). Fourteen patients (14 hips) had a good or excellent clinical result. Two patients (two hips) were classified as failures based on mHHS less than 70 (n = 1) or conversion to total hip arthroplasty (n = 1).

The authors concluded that combined surgical hip dislocation and PAO provides major deformity correction in Perthes-like hip deformities with associated acetabular dysplasia. Early clinical results suggest this technique is safe and effective, while long-term studies are needed to determine if improved long-term outcomes are associated with comprehensive deformity correction.

## Beyond the alpha angle: Alternative measurements for quantifying cam-type deformities in femoroacetabular impingement[4]

Swiss researchers have attempted to assess alternative measurements to the alpha angle as a tool for distinguishing between symptomatic and asymptomatic cam-type deformities of the femoral head.

Magnetic resonance imaging (MRI) examinations of 106 individuals (age 20-50 years) from a previous study on the alpha angle were analysed, including 53 femoroacetabular impingement (FAI) patients with cam-type deformities and 53 age-/sex-matched asymptomatic volunteers. On radially reformatted MR images two independent radiologists assessed femoral offset and femoral distance (FD) around the femoral head circumference.

The mean offset was smallest in the anterosuperior position for both readers (reader 1: 6.2 +/− 2.9 mm patients and 7.3 +/− 1.8 mm in volunteers, p = 0.002 and reader 2: 6.1 +/− 3.3 mm in patients and 7.1 +/− 2.9 mm in volunteers, p = 0.111). The mean FD was highest in the anterosuperior position for reader 1 (patients 3.3 +/− 1.4 mm; volunteers 1.7 +/− 2.2 mm; P < 0.001) and in the anterior position for reader 2 (patients 3.1 +/− 1.7 mm; volunteers 2.0 +/− 1.5 mm; P = 0.001). Overall interobserver agreement (ICC) was good (offset 0.657/FD 0.632). ROC analysis for offset measurements showed the largest area under the curve in anterosuperior position for reader 1 (0.666) and in posterosuperior position for reader 2 (0.612). For FD measurements, the area under the curve was largest in anterosuperior position for both readers (0.793/0.798).

The authors concluded that while FD measurements were superior to offset measurements and showed similar results to the alpha angle, neither FD nor offset measurements are a reliable tool for discrimination between FAI patients with cam-type deformities and asymptomatic volunteers.

## Prevalence of Femoroacetabular Impingement Imaging Findings in Asymptomatic Volunteers: A Systematic Review[5]

In a systematic review the authors aimed to determine the prevalence of radiographic findings suggestive of femoroacetabular impingement (FAI) in asymptomatic individuals.

Studies reporting radiographic, computed tomographic, or magnetic resonance imaging (MRI) findings that were suggestive of FAI in asymptomatic volunteers were included. Cam, pincer, and combined pathologic conditions were investigated.

A total of 26 studies were found to be suitable for inclusion, comprising 2,114 asymptomatic hips (57.2% men; 42.8% women). The mean participant age was 25.3 +/− 1.5 years. The mean alpha angle in asymptomatic hips was 54.1 degrees +/− 5.1 degrees. The prevalence of an asymptomatic cam deformity was 37% (range 7-100%) − 54.8% in athletes versus 23.1% in the general population. Of the 17 studies that measured alpha angles, 9 used MRI and 9 used radiography (1 study used both). The mean lateral and anterior center edge angles (CEAs) were 31.2 degrees and 30 degrees, respectively. The prevalence of asymptomatic hips with pincer deformity was 67% (range 61-76%). Pincer deformity was poorly defined (4 studies [15%]; focal anterior overcoverage, acetabular retroversion, abnormal CEA or acetabular index, coxa profunda, acetabular protrusio, ischial spine sign, crossover sign, and posterior wall sign). Only 7 studies reported on labral injury, which was found on MRI without intra-articular contrast in 68.1% of hips.

The review concluded that FAI morphologic features and labral injuries are common in asymptomatic patients. Clinical decision making should carefully analyse the association of patient history and physical examination with radiographic imaging.

## Patient-Reported Outcome (PRO) questionnaires to measure hip and groin disability in young-aged to middle-aged adults.[6]

Danish scientists conducted a systematic review to determine the recommended patient reported outcome measures for young patients with hip pathology. The methodological quality of the studies included was determined using the COnsensus-based Standards for the selection of health Measurement INstruments list (COSMIN) together with standardised evaluations of measurement properties of each PRO.

A total of twenty studies were included. Nine different questionnaires for patients with hip disability, and one for hip and groin disability, were identified. Hip And Groin Outcome Score (HAGOS), Hip Outcome Score (HOS), International Hip Outcome Tool-12 (IHOT-12) and IHOT-33 were the most thoroughly investigated PROs and studies including these PROs reported key aspects of the COSMIN checklist. HAGOS and IHOT-12 were based on studies with the least ratings of poor study methodology (23% and 31%, respectively), whereas IHOT-33 and HOS had a somewhat larger distribution (46%). These PROs all contain adequate measurement qualities for content validity (except HOS), test–retest reliability, construct validity, responsiveness and interpretability. No information or poor quality rating on methodological aspects made it impossible to fully evaluate the remaining PROs at present.

The authors concluded that HAGOS, HOS, IHOT-12 and IHOT-33 can be recommended for assessment of young-aged to middle-aged adults with pain related to the hip joint, undergoing non-surgical treatment or hip arthroscopy. At present, HAGOS is the only PRO also aimed for young-aged to middle-aged adults presenting with groin pain and is recommended for use in this population.
